# Subclinical Left Ventricular Systolic Dysfunction in Hospitalized Patients with COVID-19 by Strain: A 30-Day Echocardiographic Follow-Up

**DOI:** 10.3390/medicina59122065

**Published:** 2023-11-23

**Authors:** Pedro Morillas-Blasco, Paula Guedes-Ramallo, Nuria Vicente-Ibarra, Marina Martínez-Moreno, Andrea Romero-Valero, Antonio García-Honrubia, Elena Castilla-Cabanes, José Antonio Viedma-Contreras, Mar Masiá-Canuto, Jesús Castillo-Castillo, Sandra Santos-Martínez

**Affiliations:** 1 Department of Cardiology, Hospital General Universitario Elche, 03203 Elche, Spain; morillas_ped@gva.es (P.M.-B.); garcia_anthon@gva.es (A.G.-H.); castilla_ele@gva.es (E.C.-C.);; 2 Clinical Laboratory Unit, Hospital General Universitario Elche, 03203 Elche, Spain; 3 Infectious Disease Unit, Hospital General Universitario Elche, 03203 Elche, Spain

**Keywords:** coronavirus disease 2019, SARS-CoV-2, cardiac troponins, heart injury, strain imaging

## Abstract

*Background and Objectives*: Available studies confirm myocardial injury and its association with mortality in patients with COVID-19, but few data have been reported from echocardiographic studies. The aim of this study was to identify subclinical left ventricular dysfunction by global longitudinal strain (GLS) and its evolution in the short term in hospitalized patients with COVID-19. *Materials and Methods*: Thirty-one consecutive noncritical patients admitted for COVID-19 were included. Information on demographics, laboratory results, comorbidities, and medications was collected. Transthoracic echocardiograms were performed using a Philips Affinity 50, at the acute stage and at a 30-day follow-up. Automated left ventricular GLS was measured using a Philips Qlab 13.0. A GLS of <–15.9% was defined as abnormal. *Results*: The mean age was 65 ± 15.2 years, and 61.3% of patients were male. Nine patients (29%) had elevated levels of high-sensitivity troponin I. Left ventricular ejection fraction was preserved in all; however, 11 of them (35.5%) showed reduced GLS. These patients had higher troponin levels (median, 23.7 vs. 3.2 ng/L; *p* < 0.05) and NT-proBNP (median, 753 vs. 81 pg/mL; *p* < 0.05). The multivariate analysis revealed that myocardial injury, defined as increased troponin, was significantly associated with GLS values (coefficient B; *p* < 0.05). Follow-up at 30 days showed an improvement in GLS values in patients with subclinical left ventricular dysfunction (−16.4 ± 2.07% vs. −13.2 ± 2.40%; *p* < 0.01), without changes in the normal GLS group. *Conclusions*: Subclinical left ventricular dysfunction is common in noncritical hospitalized patients with COVID-19 (one in every three patients), even with preserved left ventricular ejection fraction. This impairment tends to be reversible on clinical recovery.

## 1. Introduction

In December 2019, an outbreak of severe acute respiratory syndrome coronavirus 2 (SARS-CoV-2) was reported in Wuhan, China [1]. Within just a few weeks, the disease, COVID-19, spread throughout the world with a high mortality rate [2].

Various published studies have shown that myocardial damage, defined as increased plasma cardiac troponin levels, is a common finding in affected patients, and that it is associated with a poorer outcome during admission and high mortality [3,4]. However, little is known about its real effect on the heart and whether increased levels of specific biomarkers reported in studies simply reflect a more systemic disease in the most severely ill patients [5]. Given the difficulty of performing an echocardiography study under strict isolation conditions, in addition to the potential risk of contagion for health care staff, the exact prevalence and nature of cardiac involvement using systematic cardiac imaging in patients with COVID-19 has been difficult to establish but of great interest. In order to analyze the cardiac involvement of SAR-CoV-2, multicenter alliances have emerged, like the ECHO_COVID research group. They assessed cardiac disorders in six hundred and seventy-seven severely ill patients affected by COVID-19 in the ICU and found that almost one-third presented abnormal LV and/or RV systolic function. Additionally, RV involvement was prevalent, with different phenotypes of RV involvement leading to different ICU mortality rates; acute cor pulmonale had the worst outcome [6,7].

Determination of the left ventricular ejection fraction using echocardiography is the most commonly used technique for the assessment of ventricular function in clinical practice. However, it may fail to detect early or subclinical abnormalities of systolic function. Measurement of the global longitudinal strain (GLS) using two-dimensional speckle-tracking echocardiography is a sensitive parameter of systolic function. Cardiomyopathy, heart failure, and evaluation of chemotherapy-induced cardiac toxicity are common clinical conditions that use left ventricular GLS to identify subclinical left ventricular dysfunction [8]. Data regarding the use of left ventricular GLS in patients with COVID-19 are limited. Accordingly, the objectives of the present study were to evaluate the characteristics and severity of myocardial injury using left ventricular GLS in patients hospitalized with COVID-19 outside the ICU, to investigate its association with plasma cardiac troponin levels, and to assess the evolution of GLS in the short term. 

## 2. Material and Methods

### 2.1. Study Population

This prospective study was based on a total of 36 consecutive noncritical adult patients admitted to an internal medicine ward at a tertiary hospital with COVID-19 infection as the primary diagnosis, within one month between 6 April 2020 and 4 May 2020, and not sick enough to require invasive mechanical ventilation. Most of them required oxygen supplementation, and some were also ventilated via CPAP. The diagnosis was confirmed by a positive result for SARS-CoV-2 in reverse-transcriptase polymerase chain reaction assay. Five patients with known heart conditions (one with hypertrophic cardiomyopathy, one with dilated cardiomyopathy, and three with ischemic heart disease) or suboptimal echocardiographic images were excluded, given the possible strain abnormalities caused by their diseases and not only due to COVID-19. 

This study fulfilled the requirements of the 1975 Declaration of Helsinki [9] and was approved by the local ethics committee (PI 48/2020). Written informed consent was requested from all participants before taking part in the study, and entry into the register did not alter the treatments administered or the actions realized. 

### 2.2. Data Collection

The demographic characteristics, clinical data, medications, laboratory findings, and cardiac biomarkers (high-sensitivity troponin I and NT-proBNP) of the participants were systematically collected during admission. Troponin levels were determined daily in the first 5 days, and the highest value was selected. Cardiac injury was defined as an increase in serum levels of high-sensitivity cardiac troponin I (hs-cTnI) above the 99th percentile (upper reference limit) [4]. In our hospital, the normal range is 5–23 ng/L. 

### 2.3. Transthoracic Echocardiography

All patients underwent transthoracic echocardiography within the first 24–48 h after admission and again 30 days later. The echocardiography was performed using an Affiniti 50 ultrasound system (Philips Medical Systems, Andover, MA, USA) by a cardiologist with expertise in echocardiographic recording and interpretation. All echocardiographic studies were bedside studies and were carried out in the designated COVID-19 ward using the same machine to minimize the risk of spread. Two-dimensional and Doppler echocardiography were performed in the standard views according to the recommendations of the European Association of Cardiovascular Imaging. All recordings included at least 3 cardiac cycles and were digitally stored for off-line analysis.

Two expert cardiologists analyzed the recorded images (averaging 3 heart cycles for statistical analysis) using a workstation running IntelliSpace Cardiovascular with the Philips QLAB 13.0 software package (Philips Andover). The volumes and ejection fraction were measured using the biplane Sympson’s method. Left ventricular GLS was assessed from 2-dimensional left ventricular apical 4-, 3-, and 2-chamber views based upon the Automated cardiac motion quantification^A.I.^ algorithm (13.0 software package from Philips (Andover)). Inter-reader and inter-vendor variabilities may compromise the clinical value of longitudinal follow-up of GLS, and that is why artificial intelligence (AI)-based, fully automated measurement of longitudinal strain has been proven more reliable compared with human interpretation [10]. Endocardial and myocardial tracings were displayed by the software, which subsequently tracked the speckles included in the region of interest frame by frame and provided a track that could be approved, rejected, or modified by the observer. With these data, the software generated measurements of both global and regional myocardial functions. 

The software provides the magnitude of longitudinal strain of 6 segments in the apical view, and the magnitude of left ventricular GLS is calculated as the average of 18 segmental strain values. In order to ensure good specificity, we selected as our lower limit of normal a GLS value of –15.9% in accordance with the literature [11]. Right ventricular strain was also evaluated using right ventricular free wall strain. A value of >−19% was used as a cut-off value for detecting right ventricular subclinical dysfunction [12]. 

The intraclass correlation coefficient was calculated to assess inter- and intraobserver reproducibility from 15 randomly selected patients using the same cine-loop for each view. The intraclass correlation coefficient for left ventricular GLS for intra- and interobserver variability was 0.91 (95% confidence interval (CI) 0.67–0.97) and 0.94 (95% CI 0.81–0.98), respectively.

### 2.4. Statistical Analysis

Data were collected onto a Microsoft Excel (Version 15—2013) spreadsheet and exported to Stata (Stata Statistical Software version 13.1, StataCorp; College Station, TX, USA). Categorical variables were expressed as absolute and relative frequencies, while continuous variables were assessed for normality using the Shapiro–Wilk test and expressed as the mean and standard deviation or median and interquartile range, as appropriate.

The means for continuous variables were compared between the distinct groups by using an unpaired Student’s T test and compared within the same group by using a paired Student’s T test when the data were normally distributed. Otherwise, the Mann–Whitney test was used. The Pearson correlation coefficient and Spearman rank correlation coefficient were used for linear correlation analysis. Proportions for categorical variables were compared using the χ2 test, although the Fisher exact test was used when data were limited. Multivariable linear regression was used to assess the relationship between predictor variables and left ventricular GLS. The final model included all variables yielding a *p*-value under 0.05 in univariable analysis, along with any other clinical variables that could plausibly influence systolic dysfunction, such as age, diabetes mellitus, dyslipidemia, and different medical treatments after discharge. 

## 3. Results 

**Figure 1:**
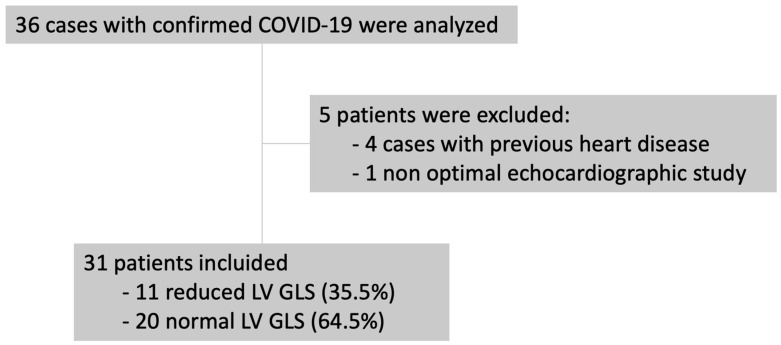
Flowchart for patient recruitment. GLS: global longitudinal strain; LV: left ventricle.

Figure 1 shows the flowchart for patient recruitment. Briefly, 4 of the initial 36 patients were excluded because of a previous history of heart disease, and 1 was excluded due to inadequate image quality. The final study population comprised 31 patients hospitalized with confirmed COVID-19. The mean age was 65 ± 15.18 years, and 61.3% were men. Myocardial damage in the form of increased troponins was detected in nine patients (29%). Table 1 shows the patients’ clinical and laboratory data.

**Figure 2:**
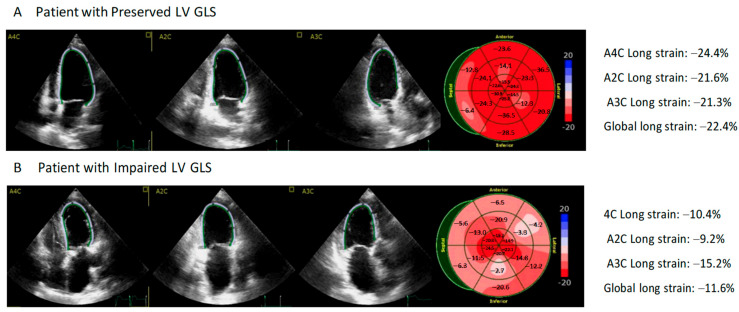
Association of Left Ventricular Global Longitudinal Strain and Prognosis in Patients with COVID-19.

All patients had a normal left ventricular ejection fraction. However, after assessing GLS, we observed that this parameter was reduced in 11 of the 31 patients (35.5%) (Figure 2). Comorbidities such as hypertension and chronic kidney disease were more common in patients with left ventricular dysfunction determined by GLS. These patients also had higher plasma hs-cTnI levels (23.7 vs. 3.2 ng/L; *p* < 0.05) and NT-proBNP levels (753 vs. 81 pg/mL; *p* < 0. 05). Moreover, 45.5% of patients with reduced GSL had elevated troponin I plasma levels. No differences were observed for plasma markers of inflammation (interleukin 6 and C-reactive protein) (Table 1 and Table 2).

Panel A shows an example of a 65-year-old patient with normal left ventricular global longitudinal strain (LV GLS) and 1.5 ng/L of high-sensitivity troponin I (hs-TnI); a longitudinal strain of −22.4% was obtained from the bull’s eye plot derived from two-dimensional speckle tracking imaging. Panel B shows results from a 72-year-old patient with impaired LV GLS and 121.4 ng/L of hs-TnI; a longitudinal strain of −11.6% was obtained from the bull’s eye plot derived from two-dimensional speckle tracking imaging.

Table 3 shows the patients’ echocardiographic characteristics according to the presence or absence of altered GLS. Patients with reduced left ventricle GLS had also lower right ventricle free wall strain. There were no differences in left ventricular filling pressure (E/e’).

**Figure 3:**
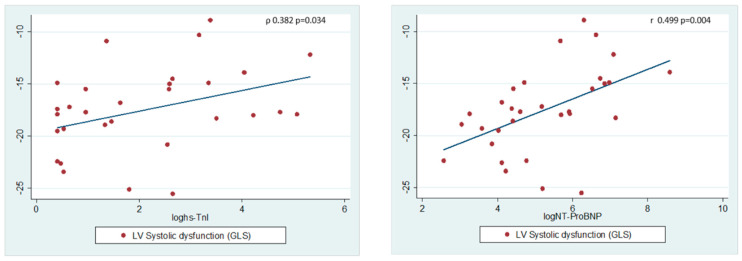
Relationship between biomarkers and left ventricular systolic function. Pearson’s correlation was used for normally distributed data, and Spearman’s rank correlation was used for data not normally distributed; two-tailed *p* < 0.05 was considered significant.

A significant and moderate positive correlation was found between GLS and plasma hs-cTnI and NT-proBNP values (Figure 3).

The multivariate analysis showed that the presence of myocardial damage, defined as an increase in hs-cTnI levels, was significantly associated with left ventricular GLS values, and patients with myocardial damage presented an abnormal 5% reduction in GLS (Table 4).

None of the patients evaluated required transfer to the intensive care unit (ICU) or died during their admission. 

**Figure 4:**
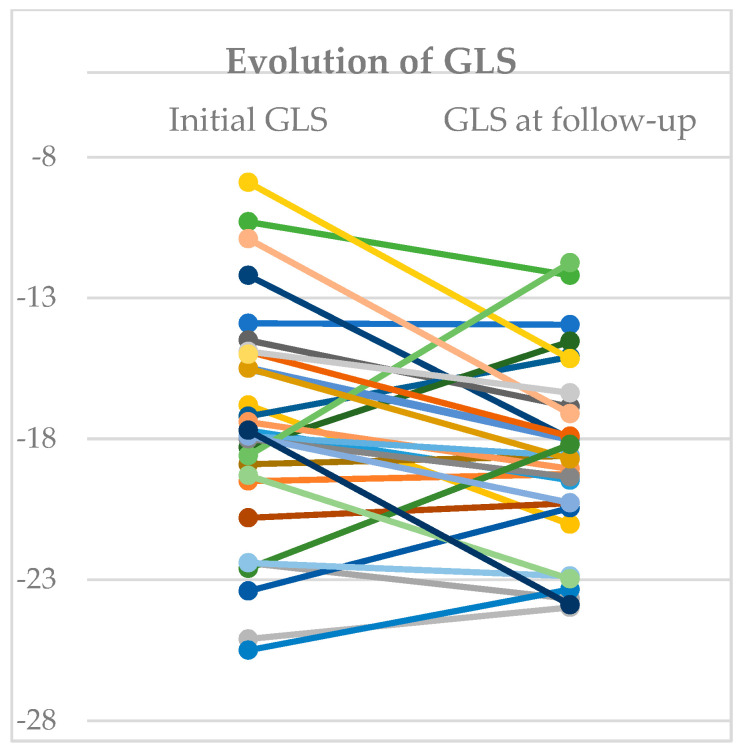
Evolution of Global Longitudinal Strain. Changes from initial global longitudinal strain and final value at follow-up. Each colour represent one patient.

At follow-up after one month, one patient from the reduced-GLS group had died, and there were no rehospitalizations. In the echocardiography performed 30 days after discharge (30 patients), there was a significant increase in GLS values in the reduced-GLS group (−16.4 ± 2.07% vs. −13.2 ± 2.40%; *p* < 0.01) (Table 5). Four of the surviving patients of this group had normalized GLS values (>−15.9%). There were no significant differences in the evolution of GLS in the group with normal systolic function during the acute phase of infection (−19.9 ± 2.73% vs. −19.8 ± 3.28%; *p* = 0.959) (Figure 4).

However, because this was an observational study on a population of a small sample of patients with COVID-19 in an internal medicine ward, our results may not reflect those for the wider population infected with the virus.

## 4. Discussion

To the best of our knowledge, this is one of the first studies to evaluate cardiac involvement based on left ventricular GLS using 2-D speckle tracking echocardiography in noncritical patients hospitalized with COVID-19. Our findings illustrate the high frequency of subclinical left ventricular dysfunction in the early stages of COVID-19 in patients with no previous history of heart disease and a preserved ejection fraction (one in three patients). Our results show that this dysfunction may be reversible on clinical recovery, since GLS had improved 1 month after the first echocardiogram in 40% of patients with impaired left ventricular function. Our data also show that lower GLS values are correlated with the magnitude of increased hs-cTnI plasma levels. 

Numerous studies have reported acute cardiac injury as an important manifestation of COVID-19. In studies published to date, acute cardiac injury, defined as an increase in cardiac troponin to the >99th percentile, affects 7% to 28% of hospitalized patients. However, this number may depend in part on the definition used and the severity of cases at the hospital where the data were recorded [3,13]. In our study, 29% of all hospitalized patients had acute cardiac injury, even though the patients’ condition was not severe and did not require admission to the intensive care unit. The percentage we report is higher, probably because, given the dynamic changes in troponin levels, we made several determinations in plasma during hospitalization and selected the highest value. Patients with high troponin levels usually present a poor clinical profile, are generally older, mainly men, and have a higher prevalence of comorbidities, including arterial hypertension, diabetes, coronary heart disease, and chronic kidney disease [14,15]. Furthermore, these values increase significantly in patients with more severe infection compared with those who have milder forms of the disease and do not require admission to the ICU [4,16]. 

Acute cardiac injury has consistently proven to be a strong negative prognostic marker in patients with COVID-19. Various studies have shown a poorer outcome in patients with increased troponin I or T: greater risk of being admitted to the ICU [17], mechanical ventilation, and more severe complications during admission, as well as respiratory distress, malignant ventricular arrhythmias, acute kidney failure, and clotting disorders, has been reported [3,13]. In a sample of 416 cases, Shi et al. [3] showed higher mortality in patients with myocardial damage (51.2% vs. 4.5%, *p* < 0.001), with an association between the magnitude of the increase in hs-cTnI levels and mortality. 

Importantly, many aspects of this increase in troponins remain undefined, including the frequency and severity of associated cardiac structural abnormalities. To date, few data have been published about how the virus affects the heart. Furthermore, not many systematic echocardiography-based studies report the exact prevalence and nature of cardiac involvement in COVID-19. In some cases, it could go undetected. The few published studies report right ventricular dysfunction and dilatation as the most common echocardiographic abnormality, with a considerable impact on prognosis of the disease [18], whereas systolic function of the left ventricle is rarely affected in patients with COVID-19.

In their retrospective study of 74 patients with COVID-19 pneumonia, Mahmoud-Elsayed et al. [19] reported frequent right ventricular abnormalities in the form of dilatation (41%) and dysfunction (27%), whereas left ventricular systolic function was normal or hyperdynamic in most patients (89%). The study, however, was subject to major limitations, mainly that the left ventricular systolic function was determined visually. A recent study of 120 patients hospitalized with COVID-19 showed that the left ventricular ejection fraction—assessed according to the Simpson biplane method—was impaired to some extent in 11% of patients, with no repercussion on patient progression during admission [20]. Similar findings were reported by Szekely et al. [21] in a series of 100 consecutive patients, among whom only 10% had a depressed ejection fraction. Among these patients, two cases were due to the presence of previous coronary disease. No differences were found in terms of cardiac injury prevalence depending on the cardiac troponin increase. The results of these studies suggest that ventricular systolic dysfunction in patients with acute COVID-19 infection is not very common, while ventricular diastolic dysfunction is more prevalent, as has been reported in other coronavirus infections [22]. 

In a multicentric study by Lassen et al. [23] of 214 patients with COVID-19, both left and right ventricular strain were reduced in comparison with those of control subjects, and left ventricular strain reduction was associated with poorer outcome. In this regard, our study provides consistent information, considering that using left ventricular GLS is an effective and reproducible assessment to quantify global systolic function of the left ventricle in patients with acute COVID-19 infection. This approach is more sensitive than the ejection fraction as a marker of cardiac dysfunction. GLS helps in the diagnosis of early and subclinical stages of myocardial involvement in different conditions such as cardiomyopathy and chemotherapy-induced cardiotoxicity before significant changes occur in ventricular function [8]. Thus, we have been able to show for the first time that asymptomatic involvement of left ventricular function is very common in a population of stable hospitalized patients (35.5%), even though these patients have a preserved left ventricular ejection fraction. The degree of ventricular dysfunction is associated with the magnitude of the increase in plasma troponin levels, as seen by the fact that patients with lower strain values have higher hs-cTnI levels. 

Previous studies suggest that myocardial tissue remodeling could precede functional remodeling, and our results point in this direction by showing early dysfunction without ejection fraction abnormalities. Among the prior studies, a small-scale study based on cardiac magnetic resonance in patients who had recovered from COVID-19 showed that 54% had myocardial edema affecting 33% of left ventricular segments and late gadolinium uptake, with no alterations in contractility [24]. Our study shows a similar left ventricular dysfunction persistence: 60% of patients still had reduced GLS at the one-month follow-up. Previous experiences with SARS showed that 40% of patients who had recovered from the disease had cardiovascular abnormalities at a 12-year follow-up, in addition to lipid abnormalities and glucose metabolism [25].

In contrast with other studies in which different degrees of right ventricular abnormalities such as dilatation or systolic dysfunction have been reported in up to 40% of patients [18,26], we found light involvement of the right ventricle during infection. This difference is mainly because these previous studies included more critical patients with acute respiratory failure who required mechanical ventilation. However, focusing on the right free wall strain, 11 of our patients (35.5%) had reduced strain values of <–19%.

Regarding the cardiac biomarkers, it has been reported that less than 50% of patients with increased levels of troponins had left ventricular systolic or diastolic dysfunction with abnormal right ventricular function. The authors of [21] suggest that the more common mechanism underlying elevated troponins in patients with COVID-19 is an acute right ventricular dysfunction due to vascular or parenchymal involvement in the lung. These findings are quite controversial since left ventricular GLS was not determined in their study, as it was in ours. We observed that patients with increased troponins have significantly more marked impairment of GLS, whereas no differences were observed in right ventricular function parameters between the two study populations. Therefore, subclinical left ventricular dysfunction may be the common mechanism in the increase in troponins in this infection.

Although the exact pathophysiology of myocardial damage in COVID-19 is not completely known, various potential mechanisms could play a role in this phenomenon. On the one hand, there is a direct invasion of cardiomyocytes by the virus, in which expression of angiotensin-converting enzyme 2 by myocytes is essential. In the same way, severe hypoxia caused by respiratory damage can lead to ischemia and oxidative stress due to the increased oxygen demand in the myocardium. On the other hand, cardiac microvascular damage can be caused by intravascular coagulation and/or by an excessive immune and inflammatory response leading to a cytokine storm and myocardial dysfunction [26,27]. It has been recently pointed out that people with chronic diseases have a higher risk of developing more severe forms of the disease. In such more ill people, lipid peroxidation is catalyzed by iron released from hemoglobin, transferrin, and ferritin, which are released by tissue acidosis and free oxygen radicals. Such ferroptosis-inducing factors can directly or indirectly affect glutathione peroxidase, resulting in a decrease in the antioxidant capacity and accumulation of lipid reactive oxygen species (ROS) in the cells, ultimately leading to oxidative cell stress and, finally, death [28]. 

In our study, SAR-CoV-2 infection was associated with a high incidence of subclinical cardiac involvement, highlighted by impairment in GLS and elevated hs-cTnI; this was reversible in most patients but it could affect prognosis as it has been seen in other myocarditis. This could mean that patients with cardiac dysfunction should have a longer follow-up to exclude former cardiac abnormalities in the long term. The application of these findings to clinical practice would involve the hypothesis that in other kinds of myocarditis with normal left ventricular ejection fraction but with subclinical left ventricular dysfunction shown by GLS and elevated hs-cTnI plasma levels, the impaired ventricular function might have a high recovery rate, as we have seen in this study with the SAR-CoV-2 virus.

Despite being based on a consecutive and homogeneous cohort of patients hospitalized for COVID-19, our main limitation is that this was a single-center observational study and was performed in a relatively small sample; because of this, the results may vary in a wider population. Furthermore, we included patients with mild disease and not with severe conditions (respiratory distress or multiorgan failure), where the systemic inflammatory response is more marked, as are its cardiac implications. Given the broad clinical variety of SARS-CoV-2 infection, which includes asymptomatic patients who are not hospitalized, our findings cannot be applied to the general population affected by COVID-19. Finally, our study does not enable us to evaluate whether these echocardiographic abnormalities can lead to chronic consequences in the long term. Therefore, careful follow-up of patients who have recovered from COVID-19 should be recommended, in order to identify the long-term implications of the cardiovascular damage. 

## 5. Conclusions

In conclusion, our study shows that subclinical left ventricular dysfunction determined by GLS is common in noncritical patients admitted for COVID-19 and is associated with hs-cTnI plasma levels. The impairment may be reversible in a significant proportion of patients (40%) in the short term. In order to provide an accurate treatment to our patients and achieve better outcomes, it would be necessary to extend our knowledge about long- and short-term cardiovascular implications, because the impairments reported in our study might have prognostic implications. Further studies are required to understand the exact cardiovascular pathophysiology suffered during COVID-19 infection.

## Figures and Tables

**Table 1:** Baseline characteristics and laboratory findings.

	Patients, No. (%)			
		LV Systolic Dysfunction (GLS)		
Characteristic	All (n = 31)	With (n = 11)	Without (n = 20)	*p* Value
Age (years), mean (SD)	65 ± 15.2	71.1 ± 11.8	61.7 ± 16	0.090
Female sex, n (%)	12 (38.7)	4 (36.6)	8 (40)	0.842
BMI (kg/m^2^), median (IQR)	27.7 (24.5–31.3)	27.5 (26.4–35.1)	25.2 (24–328.4)	0.091
**Comorbidities, n (%)**				
Hypertension	15 (48.4)	8 (72.7)	7 (35)	0.044
Diabetes mellitus	4 (12.9)	1 (9.1)	3 (15)	0.639
Dyslipidemia	10 (32.3)	4 (36.4)	6 (30)	0.717
Current smoking habit	4 (12.9)	1 (9.1)	3 (15)	0.639
Previous Stroke/TIA	1 (3.3)	1 (9.1)	0 (0)	0.181
COPD	2 (6.5)	0 (0)	2 (10)	0.278
Chronic renal failure *	3 (9.7)	3 (27.2)	0 (0)	0.014
**Treatment at admission, n (%)**				
Statins	5 (16.1)	3 (27.3)	2 (10)	0.096
Diuretics	9 (29)	5 (45.5)	4 (20)	0.227
Oral anticoagulants	4 (12.9)	2 (18.2)	2 (10)	0.030
Beta-blockers	4 (12.9)	2 (10.2)	2 (10)	0.322
ACE inhibitors/ARB	9 (29)	4 (36.6)	5 (25)	0.505

ACE: angiotensin converting enzyme; ARB: angiotensin II receptor blockers; BMI: body mass index; COPD: chronic obstructive pulmonary disease; GLS: global longitudinal strain; LV: left ventricle; SD: standard deviation; TIA: transient ischemic attack. * Glomerular filtration rate < 60 mL/min/1.73 m^2^.

**Table 2:** Laboratory findings.

	Patients, No. (%)			
		LV Systolic Dysfunction (GLS)		
Laboratory Finding, Median (IQR)	All (n = 31)	With (n = 11)	Without (n = 20)	*p* Value
Hemoglobin, g/dL	13.5 (11.4–14.2)	12.8 (9.6–14.6)	13.5 (12.7–14)	0.255
Leucocytes/µL	7350 (5450–10,240)	9880 (7820–12,500)	6830 (5200–9030)	0.380
Lymphocytes/µL	1585 (760–2380)	1020 (370–1280)	1610 (1570–2380)	0.654
C-Reactive Protein, mg/dL	22.9 (9.4–70.8)	60.9 (18.5–152)	17.2 (6.4–43.9)	0.190
IL-6, pg/mL	28.2 (8.7–180.9)	28.2 (12–347)	29.1 (3.6–139.3)	0.491
Ferritin, ng/mL	327 (151–944)	897 (200–1730)	300 (441–440)	0.534
D-Dimer, µg/mL	1.3 (0.3–4.9)	2.6 (0.4–6.3)	0.9 (0.2–2.8)	0.134
LDH, U/L	178 (145–215)	197 (168–498)	165 (138–211)	0.015
Hs-TnI, ng/L	5.1 (1.7–28.5)	23.7 (13.3–29.5)	3.2 (1.6–13.2)	0.049
Troponin positive, n (%)	9 (29)	5 (45.5)	4 (20)	0.037
NT-ProBNP, pg/mL	178 (61–681)	753 (292–1070)	81 (52–240)	0.014
Creatinine, mg/dL	0.9 (0.7–1)	0.9 (0.7–1.1)	0.9 (0.7–0.9)	0.880
Albumin, g/dL	3.9 (3.2–4.1)	3.5 (2.8–3.9)	4 (3.5–4.2)	0.262
Potassium, mEq/L	4.2 (4–4.6)	4.61(3.6–4.3)	4.3 (4.1–4.6)	0.401
Sodium, mEq/L	140 (138–142)	140 (138–140)	141 (137–142)	0.995

Hs-TnI: high-sensitivity troponin I; IQR: interquartile range; LDH: lactic acid dehydrogenase; NT-ProBNP: N-terminal pro-brain natriuretic peptide.

**Table 3:** Differences in echocardiography regarding left ventricular systolic function by strain.

	Patients, No. (%)			
		LV Systolic Dysfunction (GLS)		
Echocardiographic Data	All (n = 31)	With (n = 11)	Without (n = 20)	*p* Value
LV end-diastolic volume (mL)	61.6 (53.3–77.5)	57 (54–64.2)	65.1 50.1–85.8)	0.163
LV end-systolic volume (mL)	18.3 (13.9–30.5)	17.7 (11–22.3)	19.3 (13.9–30.9)	0.340
LVEF (%)	68.6 (65–73.2)	70.8 (74.5)	67.1 (64.1–70.6)	0.496
IVS (mm)	12 (10–13)	12 (10–15)	11 (9.5–12)	0.110
LA indexed volume (mL/m^2^)	29.6 (21.9–35.2)	34.4 (22.9–47.5)	28.3 (18.8–32.9)	0.698
E/A ratio	0.8 (0.7–0.9)	0.7 (0.6–0.8)	0.9 (0.7–1.1)	0.109
E/é ratio	9 (7.1–12.2)	9.1 (8–11.5)	8.4 (6.6–13.1)	0.942
TAPSE (mm)	21 (19–25)	20 (18–26)	22 (19–24)	0.915
RV basal diameter (mm)	33.5 (29–36)	33 (28–35)	33.5 (29–36)	0.597
RV FAC (%)	50.9 (45.8–56.1)	55.9 (50.4–66)	48.4 (45.5–55.9)	0.092
RV free wall strain (%)	−22.4 (−27.6–−15.8)	−15.2 (−20.9–−15.2)	−24.4 (−28.8–−21.2)	0.002
Anormal RV strain (%)	11 (35.5%)	7 (63.6%)	4 (20%)	0.015

FAC: fractional area change; GLS: global longitudinal strain; IVS: interventricular septum; LA: left atrium; LV: left ventricle; LVEF: left ventricular ejection fraction; RV: right ventricle; TAPSE: tricuspid annular plane systolic excursion.

**Table 4:** Multivariable linear regression to assess the relationship between predictor variables and the value of GLS.

	Coefficients B	Std. Error	*t*	95% CI	*p* Value
Age	0.613	0.055	1.11	−0.054–0.177	0.282
Troponin positive	5.008	2.333	2.15	0.141–9.875	0.044
NT-proBNP	−0.001	0.001	−0.65	−0.003–0.002	0.524
Hypertension	−3.579	2.724	−1.31	−9.261–2.103	0.204
Diabetes mellitus	−3.173	2.902	−1.09	−9.227–2.881	0.287
Dyslipidemia	1.359	1.700	0.80	−2.187–4.906	0.433
Chronic renal failure	5.899	3.071	1.92	−0.507–12.305	0.069
ACE inhibitors/ARB	3.917	2.513	1.56	−1.324–9.159	0.135
Beta-blockers	2.295	3.069	0.75	−4.108–8.698	0.463
Oral anticoagulants	0.717	3.076	0.23	−5.701–7.134	0.818

ACE: angiotensin converting enzyme; ARB: angiotensin II receptor blockers; NT-ProBNP: N-terminal pro-brain natriuretic peptide.

**Table 5:** Echocardiographic data findings at baseline and a 30-day follow-up in patients with left ventricular dysfunction in the acute stage of infection (n = 10 patients).

	Baseline	30 d	*p*
LV Systolic dysfunction (GLS)	−14.2 (−14.9–−10.9)	−19.9 (15.2–−18)	<0.001
LV end-diastolic volume (mL)	56.8 (54–61.6)	45.5 (39–79)	0.898
LV end-systolic volume (mL)	17.3 (11–19.9)	15.4 (13–21.8)	0.498
LVEF (%)	70.8 (66.3–74.5)	67.3 (59.7–71.4)	0.243
LA indexed volume (mL/m^2^)	34.8 (22.4–53.5)	34.8 (22.4–53.5)	0.846
E/A ratio	0.71 (0.55–0.85)	0.71 (0.55–0.85)	0.587
E/é ratio	9 (8–10.2)	9 (8–10.2)	0.318
TAPSE (mm)	20 (18–26)	20 (20–23)	0.529
RV basal diameter (mm)	34 (30–35)	33.5 (30–36)	0.214
RV free wall strain	−15.1 (−19.8–−10.8)	−24.3 (19.3–−27.3)	0.135

GLS: global longitudinal strain; LA: left atrium; LV: left ventricle; LVEF: left ventricular ejection fraction; TAPSE: tricuspid annular plane systolic excursion; RV: right ventricle.

## Data Availability

The data presented in this study are available on request from the corresponding author.

## Abbreviations

COVID-19	coronavirus disease 2019.
SARS-CoV-2	severe acute respiratory syndrome coronavirus 2.
GLS	global longitudinal strain.
hs-cTnI	high-sensitivity cardiac troponin I.
IL-6	Interleukin 6.
ICU	intensive care unit.
TAPSE	tricuspid annular plane systolic excursion
